# Delirium Superimposed on Dementia Strongly Predicts Worse Outcomes in Older Rehabilitation Inpatients

**DOI:** 10.1016/j.jamda.2013.12.084

**Published:** 2014-05

**Authors:** Alessandro Morandi, Daniel Davis, Donna M. Fick, Renato Turco, Malaz Boustani, Elena Lucchi, Fabio Guerini, Sara Morghen, Tiziana Torpilliesi, Simona Gentile, Alasdair M. MacLullich, Marco Trabucchi, Giuseppe Bellelli

**Affiliations:** a Department of Rehabilitation and Aged Care of the Ancelle Hospital, Cremona, Italy; b Geriatric Research Group, Brescia, Italy; c Department of Public Health and Primary Care, University of Cambridge, Cambridge, UK; d Pennsylvania State University, University Park, PA; e Indiana University Center for Aging Research and Regenstrief Institute, Inc, Indianapolis, IN; f Edinburgh Delirium Research Group, University of Edinburgh, Edinburgh, Scotland; g University of Tor Vergata, Rome, Italy; h Department of Health Sciences, University of Milano Bicocca and Geriatric Clinic, San Gerardo Hospital, Monza, Italy

**Keywords:** Delirium-superimposed dementia, dementia, delirium, mobility, institutionalization, mortality, elderly

## Abstract

**Objective:**

Delirium superimposed on dementia (DSD) is common in many settings. Nonetheless, little is known about the association between DSD and clinical outcomes. The study aim was to evaluate the association between DSD and related adverse outcomes at discharge from rehabilitation and at 1-year follow-up in older inpatients undergoing rehabilitation.

**Design:**

Prospective cohort study.

**Setting:**

Hospital rehabilitation unit.

**Participants:**

A total of 2642 patients aged 65 years or older admitted between January 2002 and December 2006.

**Measurements:**

Dementia predating rehabilitation admission was detected by DSM-III-R criteria. Delirium was diagnosed with the DSM-IV-TR. The primary outcome was that of walking dependence (Barthel Index mobility subitem score of <15) captured as a trajectory from discharge to 1-year follow-up. A mixed-effects multivariate logistic regression model was used to analyze the association between DSD and outcome, after adjusting for relevant covariates. Secondary outcomes were institutionalization and mortality at 1-year follow-up, and logistic regression models were used to analyze these associations.

**Results:**

The median age was 77 years (interquartile range: 71–83). The prevalence of DSD was 8%, and the prevalence of delirium and dementia alone were 4% and 22%, respectively. DSD at admission was found to be significantly associated with almost a 15-fold increase in the odds of walking dependence (odds ratio [OR] 15.5; 95% Confidence Interval [CI] 5.6–42.7; *P* < .01). DSD was also significantly associated with a fivefold increase in the risk of institutionalization (OR 5.0; 95% CI 2.8–8.9; *P* < .01) and an almost twofold increase in the risk of mortality (OR 1.8; 95% CI 1.1–2.8; *P* = .01).

**Conclusions:**

DSD is a strong predictor of functional dependence, institutionalization, and mortality in older patients admitted to a rehabilitation setting, suggesting that strategies to detect DSD routinely in practice should be developed and DSD should be included in prognostic models of health care.

Delirium is an acute neuropsychiatric disorder characterized by inattention, generalized cognitive impairments, and disturbances in consciousness mainly affecting older, hospitalized patients. Delirium that occurs in patients with dementia is referred to as delirium superimposed on dementia (DSD).^1^

The prevalence of DSD has been reported in acute hospitals, nursing homes, and community populations, but there are few studies in rehabilitation facilities. In the only systematic review investigating its prevalence in various care settings, of 15 studies identified, none were in postacute care rehabilitation settings.^1^ However, a high proportion of patients in acute hospitals have dementia or cognitive impairment,^2^ and a significant proportion is discharged to postacute facilities with delirium still present.^3^, ^4^, ^5^, ^6^, ^7^

Both delirium and dementia affect functional recovery, and especially affect the ability to recover walking after an acute illness.^8^, ^9^, ^10^, ^11^, ^12^, ^13^, ^14^, ^15^, ^16^, ^17^, ^18^ This also has been demonstrated in community populations.^19^, ^20^ Little attention has been given to the impact of delirium, and specifically of DSD, on functional outcomes in rehabilitation settings, despite the need to predict functional improvement as a part of the rehabilitation process. The occurrence of delirium alone has been shown in rehabilitation hospitals to be linked to worse functional outcomes^4^, ^21^ while the effect of dementia alone is still controversial.^22^ One might expect that the overlap between delirium and dementia as the overlap of delirium with depressive symptoms^23^ might indeed expose the patient to a greater risk of adverse outcomes after a rehabilitation treatment. Only one study,^3^ to our knowledge, has provided preliminary information on the association between DSD and functional status. This study found that patients with DSD were significantly more functionally impaired on admission in comparison with those with dementia alone, delirium alone, or neither of these conditions, and that DSD was a predictor of the risk of institutionalization at discharge. However, the authors did not assess the role of DSD in predicting functional recovery at discharge and did not evaluate the effect of confounding factors.

Delirium also has been shown to predict institutionalization and mortality in different clinical settings^24^ but few studies have been conducted to understand the association between DSD and these outcomes in older adults admitted to acute hospitals and rehabilitation settings.^3^, ^17^, ^18^, ^25^ DSD predicted worse functional outcomes and institutionalization in elderly patients at 1-month and up to 1-year discharge from an acute hospital, although the definition of dementia in these 2 studies^17^, ^18^ was carried out differently. One study used the IQCODE^18^ and the second one used a more rigorous approach.^17^ DSD in rehabilitation settings was an important predictor of 1-year mortality in a group of 188 older patients^25^ and of institutionalization at rehabilitation discharge in 2340 elderly patients.^3^ However, no information has been provided on the long-term effect of DSD on institutionalization in older patients admitted to a rehabilitation settings and on the importance of DSD on long-term mortality in a large sample population in these settings.

To address the paucity of data in this area, the purposes of this study were to evaluate (1) the association between DSD and functional outcomes, specifically walking recovery at discharge and at 1-year follow-up; and (2) the association among DSD, institutionalization, and mortality at 1-year follow-up in a cohort of older inpatients in a rehabilitation unit.

## Methods

This was a prospective cohort study of inpatients aged 65 and older consecutively admitted to a rehabilitation unit between January 2002 and December 2006 either after acute hospitalization or directly from home. The study was conducted in the Department of Rehabilitation and Aged Care (DRAC) at the “Ancelle della Carità” Hospital (Cremona, Italy), an 80-bed unit staffed by geriatricians; psychiatrists; neuropsychologists; nurses; and physical, speech, and occupational therapists. The characteristics of this clinical setting have been previously described.^26^ The Ethics Committee of Gerontological Sciences of the Geriatric Research Group approved the study. Informed consent was obtained from each patient at admission or an available proxy.

### Measures

Demographics included age and sex. Comorbidity was defined according to the Charlson Comorbidity Index (CCI).^27^ Admission diagnoses to the DRAC were recorded. Overall functional status was assessed with the Barthel Index (BI)^28^, ^29^ through patient and surrogate interview referring to 3 time points: (1) 1 month before the rehabilitation admission; (2) admission to the rehabilitation facility; and (3) at discharge.

Presence of delirium at the time of admission was screened for with the Confusion Assessment Method (CAM) algorithm and it was confirmed by a gold standard clinical assessment using the Diagnostic and Statistical Manual of Mental Disorders (4th edition, text revision [DSM-IV-TR]) by 3 geriatricians (G.B., F.G., R.T.) trained in delirium and dementia assessment.

The presence of dementia was ascertained during inpatient rehabilitation by a consensus of 2 out of 3 geriatricians (G.B., F.G., R.T.) and 1 out of 2 neuropsychologists (E.L, S.M.) in accordance with the Diagnostic and Statistical Manual of Mental Disorders (3rd edition, revised [DSM-III-R, 1987]) criteria using a standardized approach, including assessment of cognitive and functional capacity, reviews of previous clinical and neuropsychological charts, and scores on Mini Mental State Examination (MMSE) and/or other neuropsychological tests. The DSM-III-R criteria were used instead of the DSM-IV-TR because they do not require a differentiation between subtypes of dementia and so defines the presence or absence of dementia per se.

To specifically evaluate the presence of DSD and to differentiate it from dementia alone, each family member or caregiver was interviewed to ascertain the acute change of mental status. Additionally, when available, previous medical records, including cognitive evaluations, were also used to support the assessors on determining the acute change in mental status and the presence of inattention. A final consensus diagnosis was obtained by 2 geriatricians (G.B., F.G., R.T.) and 1 neuropsychologist (E.L., S.M.). Instances of disagreement between 2 geriatricians and a neuropsychologist were resolved by consensus among the 3 geriatricians and the 2 neuropsychologists.

### Primary Outcome

The primary outcome was that of walking dependence captured as a trajectory from discharge to 1-year follow-up. Degree of walking dependence at discharge and at 1-year follow-up was assessed using the BI walking mobility subitem. A score less than 15 (the maximum score) is robust to the presence of mobility impairment.^30^, ^31^ The BI administered by telephone has been shown to be as reliable as to direct face-to-face assessment.^32^ This primary outcome was defined a priori.

Participants were recontacted by telephone to assess walking ability at 1-year follow-up. The interviewers (F.G., R.T.) asked the patient or the caregiver to indicate the most accurate description of the participant's functional status after reading all possible answers for the BI walking subscore.

### Secondary Outcomes

Nursing home placement and mortality were ascertained through telephone interview with family members at 1 year after the discharge.

### Statistical Analysis

Demographics and clinical variables were summarized using median and interquartile range (IQR) for continuous variables or proportions for categorical variables. The independent associations between cognitive diagnosis (none, dementia, delirium, DSD) (exposure) and walking dependency at discharge and at 12 months (outcomes) were estimated using random-effects logistic regression models, with a random effect for intercepts and slopes. Specifically, dementia, delirium, and DSD were compared with the reference group (no delirium and no dementia.) Such a model allows accounts for the longitudinal effects of cognitive diagnosis on the outcome; that is, how delirium and/or dementia influence general walking dependency at discharge and the change 1 year later. Models were adjusted by age, sex, length of stay, preadmission walking impairment, place of care before admission, C-reactive protein, and CCI.^33^ These last 2 variables were transformed to accommodate the degree of positive skew. These variables were selected a priori according to their potential clinical relevance on the outcomes. In this model, patients who died in the year following discharge were excluded.

Two additional logistic regression models were used to estimate the association between cognitive diagnosis (none, dementia, delirium, DSD) and nursing home (NH) placement and mortality at 1-year follow-up. Models were adjusted for the same covariates of the random-effects logistic regression models. In the model exploring the association between DSD and NH placement, we excluded patients who died in the year following discharge.

To evaluate a possible interaction between delirium and dementia we constructed 3 additional models including an interaction term (delirium*dementia) and 2 separate variables (ie, delirium and dementia). All the other variables were the same as described previously in the random-effects logistic regression model and in the 2 logistic regression models.

All statistical analyses were performed using STATA version 12 (Stata Corp, College Station, TX).

## Results

A total of 2642 patients were consecutively admitted to the DRAC during the study period (Table 1). The patients had a median age of 77 years and most were women (73%). About half of the patients were admitted from an acute hospital (n = 1140); the remaining were either admitted from home (n = 1195) or from other rehabilitation settings (n = 307). The main admission diagnoses were orthopedic (37%) and neurologic (37%), followed by gait disturbances (18%). The prevalence of DSD on admission was 8%, and the prevalence of delirium alone and dementia alone were 4% and 22%, respectively.

**Table 1:** Characteristics of 2642 Elderly Patients Admitted to a Rehabilitation and Age Care Unit

Variable	n = 2642
Age, y, median (IQR)	77 (71–83)
Sex, female, n (%)	1908 (73)
Barthel Index preadmission, median (IQR)	92 (74–100)
Barthel Index at admission, median (IQR)	60 (36–82)
Barthel Index at discharge, median (IQR)	87 (66–97)
Place of care before rehabilitation admission, n (%)	
Home	1192 (45)
Non home∗	1450 (55)
Charlson Comorbidity Index, median (IQR)	2 (1–3)
C-reactive protein at admission, mg/dL, median (IQR)	0.7 (0.5–3.7)
Admission diagnoses, n (%)	
Orthopedic	957 (37)
Neurologic†	993 (37)
Cardiovascular	147 (6)
Respiratory	60 (2)
Gait disturbances	485 (18)
Delirium alone at admission, n (%)	110 (4)
Dementia alone at admission, n (%)	584 (22)
Delirium superimposed on dementia at admission, n (%)	213 (8)
Length of stay, d, median (IQR)	21 (16–28)

IQR, interquartile range.

∗ Either acute hospital or other rehabilitation settings.

† Including stroke, Parkinson disease, and neurologic gait disturbances.

**Fig. 1:**
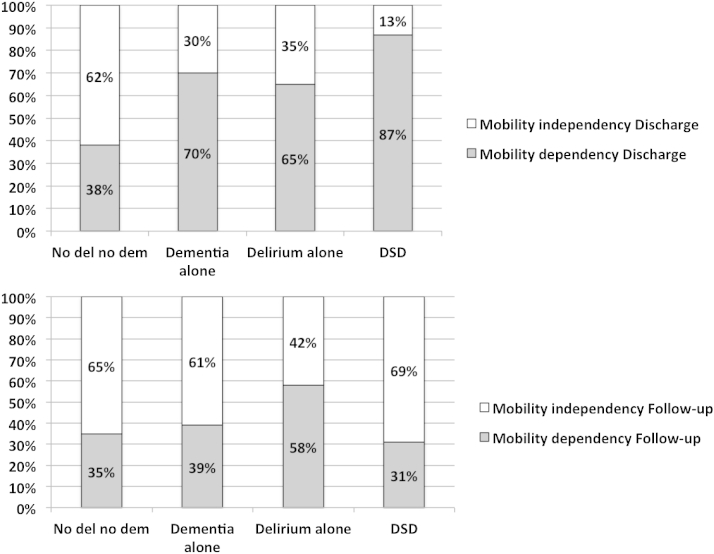
Distribution of mobility dependence at discharge and at follow-up according to the cognitive diagnosis (no delirium no dementia, delirium alone, dementia alone, DSD). In this description are excluded the 239 patients who died in the year after the discharge. Mobility dependency was defined using the Barthel Index mobility subitem score <15.

Of the patients with DSD, 87% (n = 145) and 69% (n = 115) presented with mobility dependency at the time of discharge and at 1-year follow-up, respectively (Figure 1; Appendix Table1). The distribution of mobility dependency in the dementia and delirium-alone group was similar. At discharge from rehabilitation, 92% were discharged to home, 4% to a nursing home, 2% were transferred to another rehabilitation facility, and 2% to an acute hospital. In the year after discharge, 176 patients were institutionalized (42% [n = 73] with dementia alone, 6% [n = 10] with delirium alone, 24% [n = 43] with DSD) and 239 died (42% [n = 67] with dementia alone, 5% [n = 13] with delirium alone, and 20% [n = 47] with DSD).

**Fig. 2:**
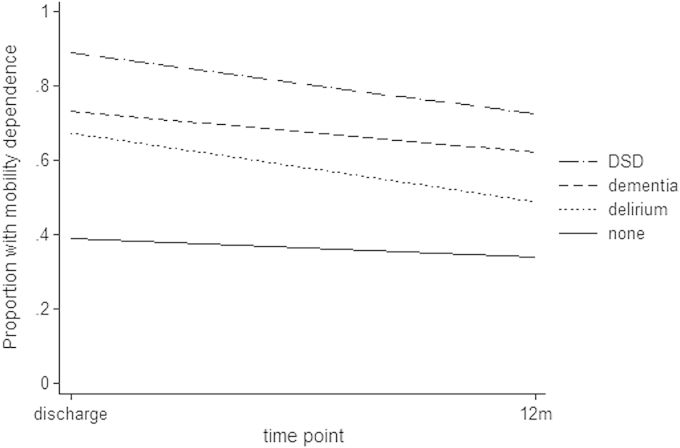
Proportion of patients with mobility impairment at rehabilitation discharge and at 1-year follow-up according to the cognitive diagnosis (none, delirium, dementia, DSD). The greatest improvements in mobility are seen in the delirium and DSD groups. The negative effect of DSD on mobility dependence is sustained at 1-year follow-up.

In the mixed-effects multivariable logistic regression model (Table 2), DSD at admission was found to be significantly associated with more than a 15-fold increase in the odds of walking dependence at discharge and at follow-up (odds ratio [OR] 15.5; 95% confidence interval [CI] 5.6–42.7; *P* < .01). Delirium alone (OR 4.3; 95% CI 2.1–8.9; *P* < .01) and dementia alone (OR 3.45; 95% CI 2.39–4.97; *P* < .01) were associated with walking dependence at discharge and at follow-up, but their effects were smaller. The evaluation of the effect of time on the odds of mobility dependency showed that (OR 0.71; 95% CI 0.58–0.87; *P* < .01) there was an overall tendency for improved mobility between discharge and follow-up. The greatest improvements in mobility dependence during the year after the rehabilitation discharge were seen in the 2 groups with DSD and delirium alone (Figure 2). Nonetheless, the negative effect of DSD on functional outcomes persisted at 1-year follow-up.

**Table 2:** Mixed-Effect Logistic Regression on the Effect of Delirium Superimposed on Dementia (DSD)∗ on Walking Dependence at Rehabilitation Discharge and at 1-Year Follow-Up†

	Odds Ratio	95% Confidence Interval	*P* Value
Dementia alone	3.45	2.39–4.97	.00
Delirium alone	4.31	2.08–8.94	.00
DSD	15.50	5.62–42.67	.00
Age	1.03	1.02–1.04	.00
Sex, female	0.83	0.67–1.02	.08
Place of care before admission (hospital)	1.11	0.91–1.37	.30
Length of stay, d	1.05	1.03–1.06	.00
Charlson Comorbidity Index	1.35	1.19–1.53	.00
Mobility dependence preadmission	8.25	6.50–10.45	.00
C-reactive protein, mg/dL	1.14	1.02–1.27	.02
Effect of diagnosis on change over time	0.71	0.58–8.71	.00
Change over time, slope	0.72	0.59–0.87	.00

∗ The diagnosis of DSD, delirium alone, and dementia alone were compared with the reference group (no delirium, no dementia).

† The independent associations between cognitive diagnosis (none, dementia, delirium, DSD) (exposure) and walking dependency at discharge and at 12 months (outcomes) were estimated using random-effects logistic regression models, with a random effect for intercepts and slopes. Specifically, dementia, delirium, and DSD were compared with the reference group (no delirium and no dementia.) Models were adjusted by age, sex, length of stay, preadmission walking impairment, place of care before admission, C-reactive protein, and comorbidity index. These variables were selected a priori according to their potential clinical relevance on the outcomes. In this model, patients who died in the year following discharge were excluded.

In the multivariable logistic regression model (Table 3), DSD at admission was significantly associated with a fivefold increase in the risk of institutionalization (OR 5.0; 95% CI 2.8–8.9; *P* < .01). Delirium alone (OR 2.4; 95% CI 1.0–5.7; *P* = .04) and dementia alone (OR 3.3; 95% CI 2.1–5.3; *P* < .01) were also significantly associated with institutionalization. Finally, DSD was associated with an almost twofold increase in the risk of mortality (OR 1.8; 95% CI 1.1–2.8; *P* = .01), whereas an association was not detected between either dementia alone or delirium alone and mortality.

**Table 3:** Association Between DSD and Nursing Home Placement and Mortality at 1-Year Follow-up

	Odds Ratio	95% Confidence Interval	*P* Value
**Nursing home placement**			
Dementia alone	3.31	2.07–5.28	.00
Delirium alone	2.41	1.02–5.66	.04
DSD	4.99	2.80–8.85	.00
Age	1.07	10.4–1.10	.00
Sex, female	0.96	0.61–1.52	.88
Place of care before admission (hospital)	1.13	0.73–1.76	.56
Length of stay, d	1.00	0.98–1.02	.92
Charlson Comorbidity Index	1.00	0.73–1.37	.97
Mobility dependence preadmission	2.55	1.54–4.21	.00
C-reactive protein, mg/dL	1.12	0.94–1.34	.21
**Mortality**			
Dementia alone	1.07	0.73–1.59	.71
Delirium alone	1.54	0.77–3.07	.22
DSD	1.76	1.10–2.80	.01
Age	1.07	1.05–1.09	.00
Sex, female	0.66	0.46–0.93	.02
Place of care before admission (hospital)	1.66	1.18–2.34	.04
Length of stay, d	0.98	0.96–0.99	.03
Charlson Comorbidity Index	1.41	1.31–1.52	.00
Mobility dependence preadmission	2.12	1.47–3.05	.00
C-reactive protein, mg/dL	1.06	1.02–1.09	.00

DSD, delirium superimposed on dementia.

Two logistic regression models were used to estimate the association between cognitive diagnosis (none, dementia, delirium, DSD) and nursing home placement and mortality at 1-year follow-up. Models were adjusted for by age, sex, length of stay, preadmission walking impairment, place of care before admission, C-reactive protein, and Charlson Comorbidity Index. In the model exploring the association between DSD and nursing home placement, we excluded patients who died in the year after discharge. The diagnosis of DSD, delirium alone, and dementia alone were compared with the reference group (no delirium, no dementia).

No statistically significant association was found for the interaction between delirium and dementia in the 3 additional models, including the interaction term delirium and dementia (data not shown).

## Discussion

This study specifically investigated the association between DSD and short- and long-term functional outcomes, including the risk of long-term mortality and institutionalization, in a large population of elderly patients admitted to a rehabilitation setting. DSD was found to be significantly associated with almost a 15-fold increase in the odds of walking dependence at rehabilitation discharge after rehabilitation training and even at 1-year follow-up. Although patients with delirium alone or dementia alone also had higher risks of worse functional outcomes at discharge and at 1-year follow-up, these risks appeared lower than in patients with DSD. DSD was also associated with a fivefold increase in the risk of institutionalization and an almost twofold increase in the risk of mortality at 1-year follow-up.

Previous studies have investigated the role of delirium on functional outcomes but they have not specifically addressed the effect of the combination of delirium and dementia.^4^, ^21^ A first study, carried out in postacute care facilities with a total population of 551 patients, found that persistent or worsening delirium on admission was significantly associated with poor functional recovery over a 1-week period both in activities of daily living (ADLs) and in instrumental ADLs.^21^ Only 5% of the sample had a preexisting diagnosis of dementia and no specific analysis addressed the effect of DSD on functional outcomes compared with patients with only delirium or dementia. The study also was limited by the fact that nurses performed delirium assessments without using a specific clinical tool to detect its presence, but used the Minimum Data Set for Post-Acute Care (MDS-PAC). The MDS-based delirium assessment has been recently reported to have limited validity.^34^ More recently, in a population of 393 elderly patients, Kiely and colleagues^4^ found that persistence of delirium was a predictor of unsuccessful functional recovery at 2-week and 1-, 3-, and 6-month follow-up. Patients who resolved their delirium by 2 weeks of postacute admission regained 100% of their preadmission functional status, whereas patients for whom delirium never resolved retained less than 50% of their preadmission functional status. Nearly a third of these patients had preexisting dementia. However, this study was performed in a postacute care setting not specifically devoted to rehabilitation. Both these studies included patients with dementia but did not specifically investigate the outcomes associated to DSD compared with the dementia or delirium-alone subgroups.

These results provide new knowledge about the possible prognostic role of DSD in patients undergoing rehabilitation, in that DSD was strongly linked to adverse outcomes. The association between DSD and adverse outcomes underlines the clinical importance of its effect. It remains uncertain if DSD is worse than delirium or dementia alone, as suggested by the differences in the ORs and as described by the distribution of mobility dependence in Figure 1. A larger study would be required to test this association adequately.

Previous investigations have reported that patients with DSD, compared with patients with dementia and delirium alone, have a twofold increased risk of being institutionalized at discharge and more than a twofold increase in the risk of mortality in the 12 months after discharge from a rehabilitation setting.^3^, ^25^ Additionally, in acute hospitals, patients with DSD compared with patients with dementia alone were exposed to a higher risk of short-, medium-, and long-term functional decline and short-term mortality.^17^, ^18^ Acutely hospitalized patients with DSD carry a significantly higher risk of institutionalization at 1-year follow-up than those with neither delirium nor dementia.^18^ In our population, the presence of DSD at the time of admission was associated with increased 1-year mortality and institutionalization rates, consistent with previous data on the effect of DSD on mortality in a smaller cohort^25^ and the reported effect of DSD on institutionalization in acutely hospitalized elderly patients.^17^, ^18^

Similar to the effect on institutionalization and mortality, in our population, DSD had an additive effect on the ability to walk independently at discharge and at 1-year follow-up for the patients with DSD and dementia alone.

The findings of worse outcomes related to DSD might be explained by reference to the pathophysiology of delirium in patients with dementia. Dementia is one of the biggest predisposing risk factors for delirium, and in this population, systemic inflammation, caused by infection, injury or surgery, is one of the major triggers.^35^, ^36^ According to the model proposed by Inouye and colleagues,^37^ severe precipitants are required to precipitate delirium in healthy populations, whereas much milder stimuli can trigger a delirious episode in patients with preexisting dementia. In these patients, even a mild infection can be the main trigger for delirium and the occurrence of DSD could lead to a more rapid cognitive decline than dementia alone, suggesting that the primary insult that causes delirium may directly exacerbate the underlying cognitive impairment.^38^, ^39^ The worsening of the cognitive impairment due to delirium could then be responsible for the worse functional outcomes seen in our study.

There are also possible clinical explanations on the association between DSD and negative outcomes. The presence of dementia alone could per se interfere with the possibility of delivering a well-organized rehabilitation intervention due to the presence of cognitive deficits, such as executive functions, memory, and attention. The literature reports inconsistent data on the implication of the presence of cognitive impairment and functional recovery after an acute illness, and in particular on the severity of cognitive impairment.^14^, ^15^, ^40^, ^41^ The coexistence of delirium and dementia is not likely to facilitate the rehabilitation process, especially in light of the worsening of the cognitive performance of patients with dementia after an episode of delirium.^19^, ^20^, ^42^ If the motor rehabilitation of patients with dementia is far from being an evidence-based discipline,^43^, ^44^ this is indeed even more evident in patients with DSD. Randomized controlled studies are warranted to provide clinicians and health care providers with specific protocols to improve the motor and cognitive rehabilitation of elderly patients with DSD.

Finally, the functional recovery between the rehabilitation discharge and the 1-year follow-up, especially in patients with DSD and delirium, might be related to a survival effect. However, the finding of greater functional recovery in the patients with delirium alone is in line with previous investigations showing that patients who actually resolve delirium have more functional recovery compared with patients without delirium or with persistent delirium.^21^ We have not assessed patients at hospital discharge and therefore we can only assume that the functional improvement is in part due to delirium resolution. These findings have not been previously shown in patients with DSD, suggesting that even in patients with dementia the excess of disability due to dementia can resolve after a rehabilitation intervention.

Our study includes a number of strengths. First, this is the first study to specifically investigate the short- and long-term effects of DSD on functional outcomes and institutionalization in a large cohort of older patients. Second, we separately considered the effect of DSD, dementia, and delirium in a setting generally underrepresented in the literature. Third, expert geriatricians collected delirium and dementia diagnoses, along with measures of functional status. Fourth, we used a valid measure to assess functional status at follow-up by telephone interview. Fifth, we achieved a 100% follow-up rate for the evaluation of functional status, mortality, and NH placement after discharge.

Limitations include the single center nature of the study. We were unable to assess duration and persistence of delirium at rehabilitation discharge and also to determine the etiology and severity of delirium. Additionally, future studies should account for the occurrence of additional episodes of delirium after the hospital discharge.

This study provides new findings on short- and long-term functional outcomes and long-term institutionalization in older patients with dementia and delirium admitted to a rehabilitation setting. The study also extends the knowledge on the long-term effect of DSD on mortality. The occurrence of DSD should be seen and considered by clinicians as an important prognostic factor. Future investigations are required to evaluate the inclusion of DSD in prognostic models for health care planning and to test intervention protocols to improve functional outcomes in patients with DSD.
