# Erratum

**DOI:** 10.1016/j.jamda.2014.05.001

**Published:** 2014-07

The authors wish to correct Figure 1 of their original study article:

Morandi A, Davis D, Fick DM, Turco R, Boustani M, Lucchi E, Guerini F, Morghen S, Torpilliesi T, Gentile S, MacLullich AM, Trabucchi M, Bellelli G. Delirium Superimposed on Dementia Strongly Predicts Worse Outcomes in Older Rehabilitation Inpatients. J Am Med Dir Assoc 2014;15:349-354.

Figure 1 was incorrect in the percentages shown in the DSD column, bottom panel. In the bottom panel the DSD percentages were actually inverted. The Mobility Independency Follow-up should be shown as 31% and the Mobility Dependency Follow-up as 69%. See the corrected Figure 1 below.

**Fig. 1:**
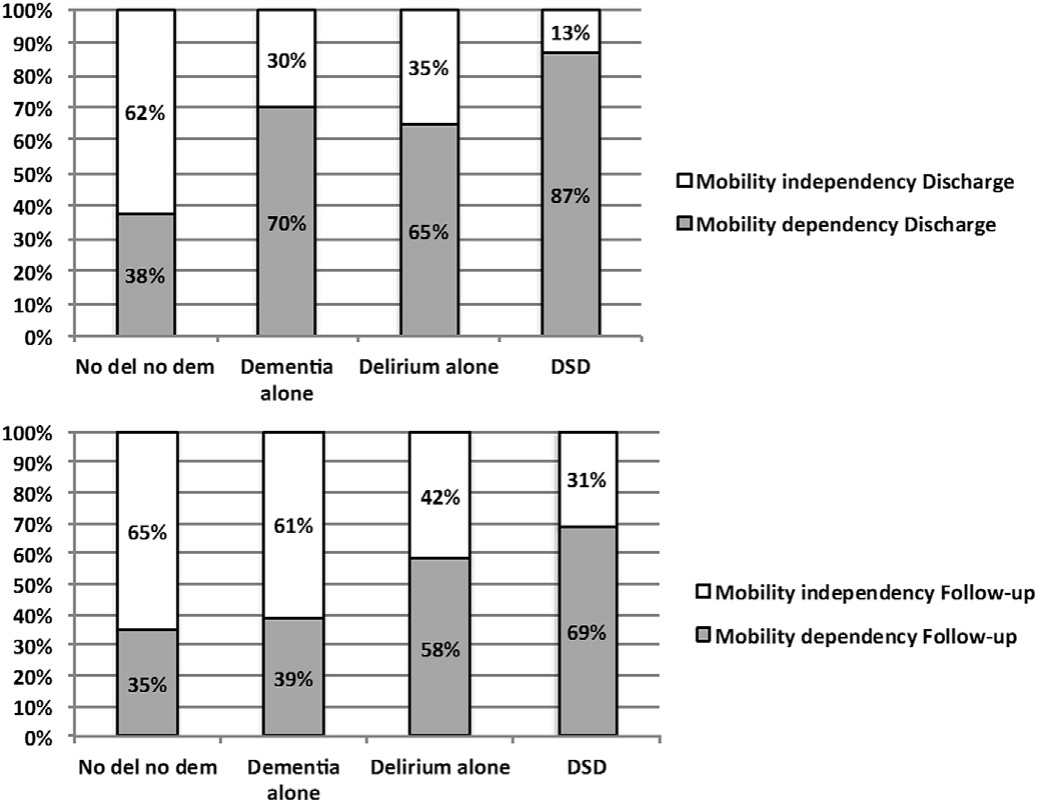
Distribution of functional status at rehabilitation discharge and at 1-year follow-up according to the cognitive diagnosis (no delirium no dementia, delirium alone, dementia alone, delirium superimposed on dementia [DSD]). The functional status was evaluated as the degree of walking dependence at discharge and at 1-year follow-up using the Barthel Index walking mobility sub-item. A score less than 15 (the maximum score) is robust to the presence of mobility dependency.^30,31^ In this description are excluded the 239 patients who died in the year after the discharge.

