# Supplementary material for: Delirium Superimposed on Dementia Strongly Predicts Worse Outcomes in Older Rehabilitation Inpatients
Source: J Am Med Dir Assoc. 2014 May;15(5):349–54. doi: 10.1016/j.jamda.2013.12.084 (PMC4004584; doi:10.1016/j.jamda.2013.12.084)
Supplement: Appendix [file mmc1.docx]

**Appendix: Supplementary data**

**eTable 1:** Distribution of mobility dependence at discharge and at follow-up according to the cognitive diagnosis (none, delirium alone, dementia alone, delirium superimposed on dementia- DSD). In this description are excluded the 239 patients who died in the year after the discharge.

|  | **Mobility dependency** | **Mobility independency** |
| --- | --- | --- |
| **At rehabilitation discharge** |  |  |
| No delirium no dementia | 627 (38.5%) | 1001 (61.5%) |
| Dementia alone | 360 (69.6%) | 157 (30.4%) |
| Delirium alone | 63 (64.9%) | 34 (35.1%) |
| DSD | 145 (87.4%) | 21 (12.6%) |
| **At 1-year follow-up** |  |  |
| No delirium no dementia | 566 (34.8%) | 1062 (65.2%) |
| Dementia alone | 315 (60.9%) | 202 (39.1%) |
| Delirium alone | 41 (42.3%) | 56 (57.7%) |
| DSD | 115 (69.3%) | 51 (30.7%) |
